# Association of Metformin Treatment with Reduced Severity of Diabetic Retinopathy in Type 2 Diabetic Patients

**DOI:** 10.1155/2018/2801450

**Published:** 2018-04-30

**Authors:** Yue Li, Christina Ryu, Metasebia Munie, Salma Noorulla, Satyesh Rana, Paul Edwards, Hua Gao, Xiaoxi Qiao

**Affiliations:** Department of Ophthalmology, Henry Ford Health System, 1 Ford Place 5D, Detroit, MI 48202, USA

## Abstract

**Purpose:**

To evaluate effects of long-term metformin on the severity of diabetic retinopathy (DR) in high-risk type 2 diabetic (T2D) patients.

**Methods:**

A retrospective chart review study was conducted involving 335 DR patients with T2D ≥ 15 years from 1990 to 2013. The severity of DR was determined by Early Treatment Diabetic Retinopathy Study scale. The associations between metformin and DR severity were evaluated. Comparison with stratification for the use of sulfonylurea and insulin was performed to identify possible confounding effects.

**Results:**

Severe nonproliferative diabetic retinopathy or proliferative diabetic retinopathy (SNPDR/PDR) was more often diagnosed in nonmetformin users (67/142, 47%) versus metformin users (48/193, 25%) (*p* < 0.001), regardless of gender and race of the patients. The odds ratio of metformin associated with SNPDR/PDR was 0.37 in all cases (*p* < 0.001), 0.35 in sulfonylurea use cohort (*p* < 0.05), 0.45 in nonsulfonylurea use cohorts (*p* < 0.01), and 0.42 in insulin use cohort (*p* < 0.01). Insulin users had a higher rate of SNPDR/PDR. Metformin had no influence on the occurrence of clinical significant diabetic macular edema.

**Conclusions:**

Long-term use of metformin is independently associated with a significant lower rate of SNPDR/PDR in patients with type 2 diabetes ≥ 15 years.

## 1. Introduction

Diabetes affects 29.3 million people in the United States [1], of which 28.5% have diabetic retinopathy (DR) [2]. About one fourth of DR will progress to the stage of severe nonproliferative diabetic retinopathy or proliferative diabetic retinopathy (SNPDR/PDR). SNPDR/PDR is usually associated with significant vision loss due to the presence of macular edema, retinal angiogenesis, vitreous hemorrhage, and retinal detachment. DR is a leading cause of legal blindness in US working-age population [3], despite the efforts in systemic metabolic control and commonly applied laser photocoagulation.

Metformin has been used to treat hyperglycemia since 1950s and becomes the first-line and the most widely used oral medication for type 2 diabetes (T2D) recently [1, 4]. Such increased usage of metformin as the preferred treatment in T2D is due to its newly identified protective effect against microvascular and macrovascular complications in diabetes patients beyond glycemic control. The landmark trial United Kingdom Prospective Diabetes Study (UKPDS) demonstrated a substantial beneficial effect of metformin on cardiovascular disease outcomes when compared with conventional diet treatment [5]. This finding was confirmed by several other independent clinical studies [6–8] and meta-analysis [9, 10] that metformin reduced risks of cardiovascular complications in diabetes patients compared with any other oral hypoglycemics or placebo, independent of its hypoglycemic effects. Further, metformin was reported to effectively improve endothelial-dependent vasodilation [11] and suppress serum markers of endothelial activation such as soluble vascular adhesion molecule-1 (sVCAM-1) and soluble intercellular adhesion molecule-1 (sICAM-1) [12, 13] in T2D patients. These evidences pointed to a potential effect of metformin on benefiting microvascular complications of diabetes such as DR. However, first-hand clinical data regarding the role of metformin in DR is scarce.

In this retrospective cross-sectional study, we assessed the relationship between long-term oral metformin and severity of DR in patients with T2D for 15 years or longer. Metformin-treated patients were found less likely to have SNPDR/PDR versus nonmetformin-treated patients, regardless of gender and race, and independent of glycated hemoglobin (HbA1c) levels. The association between metformin and a significant lower rate of SNPDR/PDR was not confounded by the use of insulin or sulfonylurea.

## 2. Methods

### 2.1. Participants

This study was a retrospective, cross-sectional study that included all patients with a diagnosis of DR and a history of T2D ≥ 15 years within Henry Ford Health System from January 1990 to September 2013. Cases were identified with an International Classification of Diseases- (ICD-) 9 diagnosis of DR. The index date of cases was the date of the most recent visit. Two groups were included in this study. One was the metformin-treated group that comprised all those who received oral metformin for the last 5 or more consecutive years. The other was the nonmetformin control group that consisted of cases that had no record of metformin use for T2D therapy. Concurrent use of insulin or other hypoglycemic agents was not excluded in both groups. Cases with intermittent metformin use, history of retinal detachment, and coexistence of other retinal vascular disorders, such as retinal vein occlusion and wet-type age-related macular degeneration, were excluded. A history of T2D ≥ 15 years and metformin treatment ≥ 5 years were chosen because prevalence of visual complication was notably increased when duration of diabetes exceeds 15 years [14], and it takes years for the effect of medication to become apparent. The study adhered to the tenets of the Declaration of Helsinki and was approved by the Institutional Review Board of Henry Ford Health System.

### 2.2. Measurements

From each case, the following information were collected: (1) demographic features, including age, gender, and race; (2) general information of T2D progression and management, such as date of T2D diagnosis, 5-year HbA1c levels (including the lowest, median, and highest values), and medications for T2D (including metformin, insulin, and other hypoglycemic agents); (3) grading of DR and prior DR treatments such as laser photocoagulation and intravitreal antivascular endothelial growth factor (VEGF) agents; and (4) ophthalmic history such as retinal vein occlusion and age-related macular degeneration.

Diagnosis of DR was made by a staff retinal specialist. The grading of DR severity and the presence of clinically significant macular edema (CSME) were determined according to the Early Treatment Diabetic Retinopathy Study (ETDRS) grading standards [15, 16]. All cases were assigned into either mild or moderate NPDR or SNPDR/PDR accordingly. If both eyes had DR, the severity would be determined due to the grade in the worse eye.

The rate of SNPDR/PDR was compared between the metformin group and the nonmetformin control group, in all cases as well as in subgroups categorized according to sex and race. The odds ratio (OR) of SNPDR/PDR was calculated to assess the association between hypoglycemic treatment and severity of DR in all cases as well as in treatment-stratified cohorts.

### 2.3. Statistics

Statistical analysis was performed using SAS software, version 9.2 (SAS Institute Inc., USA). Group difference of categorical variables was determined using standard chi-square test in the presence of nonsparse data, and Fisher exact test in the presence of sparse data. Sparsity was defined as the presence of expected cell counts less than 5. Group comparisons of numeric variables were made using two-sample *t*-test in the presence of distributional normality, and Wilcoxon rank sum test in the presence of distributional nonnormality. The ORs and 95% confidence intervals were obtained from logistic regression analysis, and the statistical significance of OR was assessed by chi-square test or Fisher's exact test. Significance was considered when *p* < 0.05.

## 3. Results

### 3.1. Demographic Features and Clinical Characteristics in the Metformin and Nonmetformin Groups

This retrospective cross-sectional study included 335 DR cases with T2D for 15 years or longer. Of all the cases, 193 (58%) had used metformin for at least 5 consecutive years and 142 (42%) had no record of metformin use. Demographic features were comparable between the two groups regarding age, gender, and race (Table 1). The duration of T2D was 15.1 ± 6.7 in the metformin group and 15.7 ± 7 in the nonmetformin group (*p* = 0.39; Table 1). The highest, lowest, and median levels of HbA1c in the most recent 5 years were well matched between the metformin group and the nonmetformin control group (*p* = 0.40, 0.81, and 0.58, resp.; Table 1).

Fewer percentage of patients used insulin in the metformin group than that in the nonmetformin group (141 of 193, 73% versus 132 of 142, 93%) (*p* < 0.001; Table 1). On the contrary, 142 of 193 (74%) metformin users also used sulfonylurea, whereas only 54 of 142 (38%) nonmetformin users had sulfonylurea treatment (*p* < 0.001; Table 1). Only 9 patients used other oral hypoglycemic agents such as thiazolidinedione, glucagon-like peptide-1 receptor agonist, and DPP-4 inhibitors.

Local therapies for DR including focal/grid laser photocoagulation, pan-retinal photocoagulation (PRP), and intravitreal anti-VEGF injection were applied in both groups. Significantly fewer patients in the metformin group received PRP versus the nonmetformin group (48 of 193, 25% versus 61 of 142, 43%) (*p* = 0.001; Table 1). There was no difference in the use of focal/grid laser photocoagulation or anti-VEGF agents between the two groups (Table 1).

### 3.2. Long-Term Metformin Treatment Was Associated with Significantly Reduced Severity of DR

**Figure 1:**
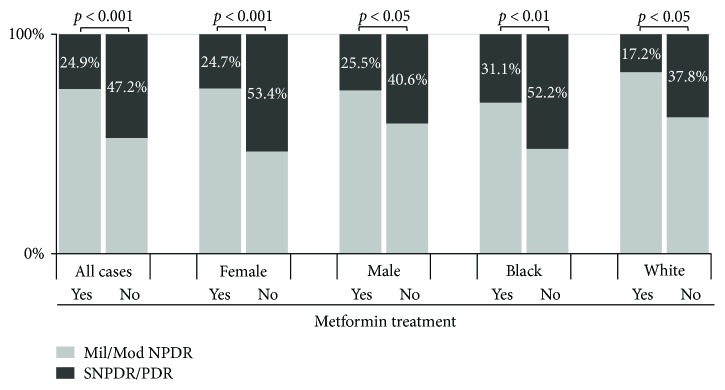
Long-term metformin treatment was associated with significantly reduced rate of SNPDR/PDR in type 2 diabetes patients. 25% of 193 metformin users were found to have SNPDR/PDR, while 47% of 142 cases that never used metformin had SNPDR/PDR (*p* < 0.001). A similar significantly lowered rate of SNPDR/PDR in metformin users versus nonmetformin users was also observed in female (25% versus 53%, *p* < 0.001) and male (26% versus 41%, *p* < 0.05), as well as in black (31% versus 52%, *p* < 0.01) and white (17% versus 38%, *p* < 0.05) subgroups. Mil/Mod NPDR: mild and moderate nonproliferative diabetic retinopathy; SNPDR/PDR: severe nonproliferative diabetic retinopathy/proliferative diabetic retinopathy.

SNPDR/PDR was diagnosed among 48 (25%) of the patients who used metformin for 5 years or longer, compared with 67 (47%) of those who never used metformin (*p* < 0.001; Table 2). The rest of the patients in both groups (53% of the nonmetformin group and 75% of the metformin group) were diagnosed with mild/moderate NPDR at the time of our study. When all cases were assessed, the OR of SNPDR/PDR in metformin users was 0.37 (95% CI, 0.23–0.59) (*p* < 0.001; Table 2), implying a 63% reduction of SNPDR/PDR associated with metformin treatment. Gender and racial subgroup analysis further revealed that longer-term metformin was correlated with significantly lower rate of SNPDR/PDR in both female and male, as well as in black and white subgroups (Figure 1).

### 3.3. Metformin-Associated Less Severe DR Was Not Confounded by the Use of Sulfonylurea or Insulin

Since there was notable difference in the use of insulin and sulfonylurea between the two groups (both *p* < 0.001; Table 1), association of these hypoglycemic therapies with the occurrence of SNPDR/PDR was evaluated. As summarized in Table 2, 54 of 197 (27%) sulfonylurea users had SNPDR/PDR, compared with 63 of 138 (46%) among nonsulfonylurea users. The OR was 0.45 (95% CI, 0.28–0.71) (*p* < 0.001; Table 2), which means a lower possibility of SNPDR/PDR in sulfonylurea-treated patients. 107 of 273 (39%) insulin-treated patients were diagnosed with SNPDR/PDR, while 8 of 62 noninsulin users (13%) had SNPDR/PDR. The OR of SNPDR/PDR by insulin was 4.35 (95% CI, 1.99–9.50) (*p* < 0.001; Table 2). Therefore, insulin was correlated with a higher rate of SNPDR/PDR, which was opposite to that of metformin.

**Figure 2:**
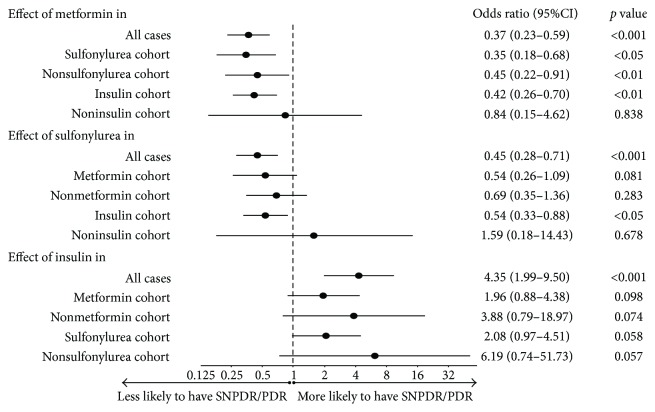
Odds ratios that indicate the association between the rate of SNPDR/PDR and hypoglycemic therapy in all cases and within each stratum of metformin, sulfonylurea, and insulin users. Metformin- or sulfonylurea-treated patients were significantly less likely to have SNPDR/PDR when all cases were assessed, while insulin users were more likely to have SNPDR/PDR versus those did not use insulin. When stratified for the use of each hypoglycemic agent, metformin showed independent association with lowered frequency of SNPDR/PDR within each stratums of hypoglycemic treatment, with statistical significance in sulfonylurea users, nonsulfonylurea users, and insulin users. However, sulfonylurea-associated significantly lower rate of SNPDR/PDR was only observed in insulin users. Insulin was associated with higher rate of SNPDR/PDR, which persisted in all the four cohorts stratified by metformin or sulfonylurea. Three of the four ORs had borderline *p* values. OR: odds ratio; SNPDR/PDR: severe nonproliferative diabetic retinopathy/proliferative diabetic retinopathy.

Further, comparison with stratification for the hypoglycemic treatments was performed to identify possible confounding effects. The association between metformin and lowered frequency of SNPDR/PDR was persistent in sulfonylurea cohort [OR 0.35 (95% CI, 0.18–0.68), *p* = 0.03], nonsulfonylurea cohort [OR 0.45 (95% CI, 0.22–0.91), *p* = 0.001], and insulin cohort [OR 0.42 (95% CI, 0.26–0.7), *p* = 0.001] (Figure 2). There was a similar trend of less SNPDR/PDR associated with metformin use in noninsulin cohort, but not statistically significant [OR 0.84 (95% CI, 0.15–4.62), *p* = 0.838] (Figure 2). These data indicated the association between metformin, and a lower rate of SNPDR/PDR was independent of sulfonylurea, and likely independent of insulin as well.

When stratified for the use of metformin, sulfonylurea treatment lost its significant association with reduced rate of SNPDR/PDR [OR 0.54 (95% CI, 0.26–1.09) and 0.69 (95% CI, 0.35–1.36), *p* = 0.081 and 0.283 for metformin and nonmetformin cohorts, resp.]. The effect of sulfonylurea was divergent when stratified for the use of insulin, with a lower rate of SNPDR/PDR in insulin cohort [OR 0.54 (95% CI, 0.33–0.88), *p* = 0.013] but a higher rate in noninsulin cohort [OR: 1.59 (95% CI, 0.18–14.43), *p* = 0.678] (Figure 2). Therefore, sulfonylurea's effect on DR severity was actually modified by the use of metformin or insulin.

It was interesting to note that patients who used insulin were more likely to have SNPDR/PDR in cohorts stratified by either metformin or sulfonylurea [OR 1.96 (95% CI, 0.88–4.38), *p* = 0.098 for metformin users; OR 3.88 (95% CI, 0.79–18.97), *p* = 0.074 for nonmetformin users; OR 2.08 (95% CI, 0.97–4.51), *p* = 0.058 for sulfonylurea users; and OR 6.91 (95% CI, 0.74–51.73), *p* = 0.057 for nonsulfonylurea users] (Figure 2). Three of the four *p* values approached the borderline of significance, indicating a noteworthy association between insulin and higher rate of SNPDR/PDR.

### 3.4. Metformin Had No Effect on the Development of CSME

Focal/grid laser photocoagulation was the major form of therapy for CSME before anti-VEGF therapy was available. CSME requiring focal/grid laser was identified in 83 of 193 (43%) cases among metformin users, and 69 of 142 (49%) cases among nonmetformin users. No significant difference was found between the two groups (*p* = 0.33).

## 4. Discussion

It is known from the United Kingdom Prospective Diabetes Study (UKPDS) that metformin significantly reduced the risk of many diabetes-related macrovascular and microvascular events when compared with other hypoglycemic therapies in diabetic patients [5]. As one of several endpoints of UKPDS, metformin lowered the risk of DR progression in overweight diabetic patients when compared with diabetic diet treatment. Inspired by UKPDS but different from it, this study focused on whether metformin influences DR severity in high-risk diabetic patients, that is, those with 15 years or longer history of T2D. We found a significant association between oral metformin for ≥5 years and less severe DR in these patients, which was not companied by a different HbA1c level. Further, this association persisted across the gender and racial subgroups and was independent of concurrent insulin or sulfonylurea treatment. The use of insulin was associated with more severe DR in our study.

### 4.1. Link between Metformin Treatment and Reduced DR Severity

As a retrospective study, we were unable to control the use of insulin and sulfonylurea among the patients. A higher percentage of nonmetformin users received insulin treatment versus that of metformin users (93% versus 73%). On the contrary, more patients of the metformin group received sulfonylurea compared with those of the nonmetformin group (74% versus 38%). Insulin therapy is generally advocated when oral medications are insufficient in controlling blood glucose. It is likely that nonmetformin users had poorly control glycemia at some point, which led to prescription of insulin and might contribute to a higher risk of DR in this group. The homogeneity in the duration of diabetes and 5-year HbA1c levels among the two groups helped provide a relatively equal comparison regarding the risk factors of DR in this study, since these two clinical parameters are believed to be the most important factors that impact DR progression [14, 17]. However, it is also important to note that tight glycemic control and a lower HbA1c have minimal effect on DR in T2D after prolonged follow-up, as evidenced by large-scale and long-term clinical studies including a 4.1-year ADVANCE study [18], a 5.6-year VADT trial [19], and a 10-year follow-up of UKPDS [20]. We assume that the different rates of SNPDR/PDR between the two groups were not simply a mirror of differently controlled blood glucose but rather reflected direct regulation on the pathogenic risk factors of DR by metformin.

The initiation of DR was associated with hyperglycemia, nonenzymatic glycosylation, and local oxidative and inflammatory stresses [21], while the progression of DR to the proliferative phase was more prominently a consequence of retinal inflammation, endothelial activation, and neovascularization [22–24]. The inflammatory cytokine ICAM-1 was one of the most studied molecules in DR pathogenesis. ICAM-1 was upregulated in the retina and vitreous of DR patients [25, 26] and diabetes rodent models [27]. ICAM-1 knockout or pharmaceutical blockade attenuated DR features such as retinal leukostasis and vascular leakage in diabetic animals [24, 27]. The assumption that metformin could directly modulate molecular events in DR progression, rather than through controlling blood glucose, was supported by clinical evidences that metformin significantly reduced plasma ICAM-1 in diabetes patients [12, 13]. In addition, we have recently reported that long-term metformin treatment effectively reduced multiple inflammatory cytokines and their correlations in the vitreous of DR patients, including ICAM-1 and interleukins [26]. Experiments in endothelial cells and STZ-induced diabetes mice by us and others also identified significant attenuation of ICAM-1 and MCP-1 levels, as well as inflammatory responses by metformin [28, 29]. The antiangiogenic activity of metformin was demonstrated in various endothelial cell lines as well as retinal neovascularization mouse models [29, 30].

One major molecular target of metformin is AMP-activated protein kinase (AMPK), a crucial cellular energy sensor that regulates lipid and glucose metabolism. Activation of AMPK was intimately associated with metformin's action in regulating the metabolic processes [31] and the inflammatory responses [28]. The association between metformin and reduced severity of DR in T2D patients could be explained by metformin-induced restoration of energy balance in the retina through activation of AMPK. However, AMPK may not account for all actions of metformin, as demonstrated by the preserved metabolic effect of metformin in liver-specific AMPK-deficient mice [32]. The regulation on endothelial inflammatory and angiogenic responses by metformin also has been shown through both AMPK-dependent and AMPK-independent mechanisms [29, 33].

Metformin did not change the rate of CSME in this study. Macular edema is mediated in substantial part by VEGF. Currently, the most successful treatment of macular edema is intravitreal injection of anti-VEGF agents [34]. Although metformin was weakly correlated with downregulation of hypoxia-inducible factor-1*α*/VEGF induced by insulin and insulin-like growth factor [35], this effect was insufficient to control the symptomatic diabetic macular edema.

### 4.2. Effects of Sulfonylurea and Insulin on DR Severity

Comparison with stratification for use of insulin or sulfonylurea treatment confirmed that the association between metformin and reduced DR severity was independent of either hypoglycemic therapy. On the other hand, although sulfonylurea was also correlated with a lower rate of SNPDR/PDR in all cases, this correlation disappeared in cohorts stratified by the use of metformin. Since 74% of sulfonylurea users also used metformin in this study, it is possible that the effect of sulfonylurea on DR severity was modified by metformin. Our data previously supported findings by the ADVANCE trial that sulfonylurea does not prevent or reduce DR progression despite reaching the glucose control goals in T2D patients with vascular complications or risk factors of vascular diseases [36].

In contrast to metformin and sulfonylurea, insulin was associated with a higher rate of SNPDR/PDR in all patients with an OR of 4.35. Stratified analysis reduced the level of significance but did not reverse this trend, with the OR values varied from 1.96 to 6.91. Similar to what we found, some other clinical studies also associated insulin with increased risk of DR in T2D patients [37, 38]. There were divergent findings about the effect of insulin on DR. UKPDS suggested that intensive blood glucose control with insulin could reduce risk of DR in newly diagnosed diabetes [39]. A Veterans Affairs Cooperative Study found that insulin had no effect on cardiovascular event or retinopathy in patients with established T2D [40]. Another study in diabetic mice associated insulin with increased retinal vascular permeability [41]. The exact impact of insulin on DR remains to be studied.

### 4.3. Strengths and Limitations of This Study

Our finding that long-term metformin treatment was associated with significantly reduced rate of SNPDR/PDR was supported by previous clinical and basic research reports on the protective effects of metformin against microvascular complications of diabetes. In particular, this study adds first-hand clinical data on the glycemic-independent effect of metformin on the severity of DR in patients with established T2D. Although there was an imbalance in the use of insulin and sulfonylurea between the two groups, we used stratified comparison to adjust for potentially confounding effects of both agents. The consistence of the crude and adjusted OR for metformin treatment helps to rule out the confounders. The difference between the crude OR and adjusted OR for sulfonylurea suggested that its effect was modified by metformin and insulin. Insulin exhibited an opposite effect on DR severity compared with metformin and sulfonylurea. As a single-centered, retrospective, cross-sectional study, we could only control a few most prominent risk factors of DR. Unmeasured confounding may remain, such as blood pressure, blood lipid, and degree of microproteinuria. Future large-scale prospective studies are warranted for a better understanding of how metformin influences the progression of DR in T2D patients.

## 5. Conclusion

Our results should be interpreted as a significant association between long-term metformin treatment and reduced severity of DR in patients with established T2D. This association exists in the lack of a different HbA1c level, persists across the gender and racial cohorts, and is not confounded by sulfonylurea or insulin treatment. Insulin users were more likely to have severe DR versus noninsulin users. Metformin might be used for the purpose of reducing DR progression in patients with long history of T2D. Our investigation is a conceptual study which calls for large-scale future studies for a full evaluation of the exact role of metformin in the progression of DR.

## Figures and Tables

**Table 1:** Demographic features and clinical characteristics of the patients.

Characters	Nonmetformin treated (*n* = 142)	Metformin treated (*n* = 193)	*p* value
Age (years)	74.2 ± 9.6	73.8 ± 10.6	0.73
Gender—number (%)			
Male	69 (49)	99 (51)	0.67
Female	73 (51)	94 (49)	
Race—number (%)			
Black	90 (63)	104 (54)	0.06
White	45 (32)	81 (42)	
Other	7 (5)	8 (4)	
Duration of diabetes (years)	15.7 ± 7	15.1 ± 6.7	0.39
HbA1c (%)			
5-year low	6.9 ± 1.1	7 ± 1.3	0.40
5-year high	9.4 ± 2	9.5 ± 1.8	0.81
5-year median	8.2 ± 1.3	8.2 ± 1.4	0.58

Treatment for diabetes—number (%)			
Insulin			
Yes	132 (93)	141 (73)	**<0.001** ^∗^
No	10 (7)	52 (27)	
Other oral hypoglycemic agent			
Sulfonylurea	54 (38)	142 (74)	**<0.001** ^**#**^
Other oral hypoglycemic agents	3 (2)	6 (3)	
No other oral hypoglycemic agents	85 (60)	45 (23)	

Treatment for diabetic retinopathy—number (%)			
Focal/grid laser photocoagulation			
Yes	64 (45)	73 (38)	0.22
No	78 (55)	120 (62)	
Pan-retinal photocoagulation			
Yes	61 (43)	48 (25)	**0.001** ^∗^
No	81 (57)	145 (75)	
Intravitreal anti-VEGF reagent			
Yes	12 (8)	23 (12)	0.37
No	130 (92)	170 (88)	

^∗^ *p* < 0.05, chi-square test; ^#^*p* < 0.05, Fisher exact test.

**Table 2:** Logistic regression analysis between patients with mild/moderate NPDR and those with SNPDR/PDR for variables associated with the use of metformin, insulin, or sulfonylurea.

Diabetes treatment	Severity of DR			
	Mil/Mod NPDR number (%)	SNPDR/PDR number (%)	OR (95% CI)	*p* value
Effect of metformin				
Nonmetformin users (*n* = 142)	75 (53)	67 (47)	**0.37 (0.23–0.59)**	**<0.001** ^∗^
Metformin users (*n* = 193)	145 (75)	48 (25)		
Effect of insulin				
Noninsulin users (*n* = 62)	54 (87)	8 (13)	**4.35 (1.99–9.50)**	**<0.001** ^∗^
Insulin users (*n* = 273)	166 (61)	107 (39)		
Effect of sulfonylurea				
Nonsulfonylurea users (*n* = 138)	75 (54)	63 (46)	**0.45 (0.28–0.71)**	**<0.001** ^∗^
Sulfonylurea users (*n* = 197)	143 (73)	54 (27)		

DR: diabetic retinopathy; Mil/Mod NPDR: mild/moderate nonproliferative diabetic retinopathy; SNPDR/PDR: severe nonproliferative diabetic retinopathy/proliferative diabetic retinopathy; OR: odds ratio; ^∗^*p* < 0.05 (logistic regression analysis).
